# The Nation and the Family: The Impact of National Identification and Perceived Importance of Family Values on Homophobic Attitudes in Lithuania and Scotland

**DOI:** 10.1007/s11199-016-0641-y

**Published:** 2016-06-04

**Authors:** Juliet R. H. Wakefield, Monika Kalinauskaite, Nick Hopkins

**Affiliations:** 1 Division of Psychology, School of Social Sciences, Nottingham Trent University, Nottingham, NG1 4BU UK; 2 Psychology, School of Social Sciences, University of Dundee, Dundee, DD1 4HN UK

**Keywords:** Homosexuality (Attitudes toward), Sexuality, Nationalism, Social identity, Family

## Abstract

The meanings attached to the nation can be consequential for group members’ attitudes and beliefs. We examined how national identity definition can influence the extent of individuals’ homophobia with 159 Lithuanian and 176 Scottish university students who completed a questionnaire which measured their national identification, homophobia, and the extent to which they felt traditional family values were central to their nation’s identity. Consistent with nation-wide differences in the significance given to the family, Lithuanian participants perceived family values to be more important for their national identity and expressed higher levels of homophobia than did Scottish participants. Moreover, the relationship between level of national identification and homophobia was stronger in Lithuania than in Scotland. Analyses revealed that the perceived importance of family values helped explain the difference between homophobia levels in Lithuania and Scotland. In both sites we found an indirect effect of national identification on homophobia via the perceived importance of family values, but this effect was significantly stronger for Lithuanian participants. These findings illustrate the ways in which identification with the nation is relevant to attitudes concerning sexuality, and how this varies according to national context. Our work indicates that LGBT rights campaigns should be informed by the knowledge that homophobia may be perpetuated by national valorisation of the family.

“A family is the foundation for a society and country”

Article 38.1 of the Lithuanian Constitution

The negative effects of homophobia on individuals, families, and communities are well-established. Homophobia can impact hiring decisions (Horvath and Ryan 2003), and the use of homophobic epithets can undermine the fair allocation of resources (Fasoli et al. 2015). In turn, sexuality-related prejudice can reduce self-esteem and encourage negative self-perceptions (Pitman 1999), as well as guilt around sex and sexual issues (Rowen and Malcolm 2003). Moreover, homophobia may contribute to depression, anxiety, substance use, self-harm, and suicidal thoughts (Chard et al. 2015; Diaz et al. 2001; Herek and Garnets 2007; Stoloff et al. 2013; Symons et al. 2014). Analyses of the predictors of homophobia highlight the roles of fundamentalist religious belief (Hildebrandt 2015; Nagoshi et al. 2008; Whitley 2009), the endorsement of conservative ideologies such as Right-Wing Authoritarianism and Social Dominance Orientation (Basow and Johnson 2000; Herek 2000; Leibold and Kühnel 2012; Whitley and Ægisdottir 2000), low levels of general education and sexuality-specific education (Chonody et al. 2009; Lambert et al. 2006), lack of opportunity for inter-group contact encounters (Smith et al. 2009), and wider cultural beliefs and understandings regarding homosexuality (Dawes 2015; DePalma and Jennett 2010). The significance of such cultural beliefs is illustrated by temporal and geographic differences in attitudes to homosexuality (Smith et al. 2014). In the present study we focus on one such cultural factor: the role of national identity-related beliefs. Specifically, we report analyses of how individuals’ level of national identification predicts homophobia in two national settings that differ in the significance given to the family as a basis for national identity: Lithuania and Scotland.

## National Identity and Sexuality

The nation is one of the most significant categories in contemporary politics. Nations can be conceptualised as “imagined communities” (Anderson 1991). They are imagined in the sense that one never knows all the other members of the community, yet individuals can still have a strong sense of connection with their fellow nationals. They are also imagined in the sense that individuals represent to themselves the values and characteristics associated with the nation, and they can come to think and act in terms of these characteristics (Reicher and Hopkins 2000). Feminist researchers have contributed much through exploring how such representations of the national community draw upon constructions of gender (McClintock 1993; Nagel 1998; Yuval-Davis 1997; Yuval-Davis and Anthias 1989).

For example, the symbolic signifiers of national difference, as well as virtue, honor, shame, and duty are routinely gendered and based on particular conceptions of male and female sexuality. In turn, many representations of national identity draw upon familial imagery. As McClintock (1993, p. 64) explains, this implies an “*organic unity* of interests” (original emphasis) which promotes a sense of horizontal community. It also implies a natural social division between men and women, and contributes to the close association between nationalist ideology and normative heterosexuality. Moreover, historical analysis shows how the emergence of modern conceptualisations of the nation were infused with bourgeois concerns over respectability and that this resulted in the celebration of heterosexuality as the bedrock of the nation, with homosexuality condemned as a nation-threatening perversion (Mosse 1985). This history has led scholars to develop the concept of heteronationalism to describe nations in which heterosexuality is perceived (and celebrated) as normative, and non-heterosexuality is perceived (and condemned) as deviant (Lazarus 2011).

## National Differences in Attitudes to Sexuality

Although constructions of national identity and sexuality are intertwined, the nature of this relationship is context-dependent. In an integrative analysis of various international surveys (e.g., the International Social Survey Program, the World Values Survey, the European Social Survey, and the Eurobarometer), Smith et al. (2014) identified increasing acceptance of non-heterosexual individuals, yet also highlighted significant cross-national differences, with ex-Communist European states being characterised by much less liberal attitudes. (Support for gay rights in these states falls below the European Union average; Mole 2011.) Although various individual-level factors contribute to such cross-national differences (e.g., individuals’ educational level, religiosity, or socio-economic status), country-level variables also play a role (Smith et al. 2014). These include the country’s level of economic prosperity: Prosperity allows citizens to shift their attention to non-material concerns relating to social values such as freedom of self-expression (Inglehart 1987). They also include the country’s level of existential security: Greater security tends to be associated with greater tolerance of pluralism (Inglehart and Welzel 2005).

Such country-level factors can also impact upon the construction of national identity. For example, in his review of homophobia in Eastern Europe, Mole (2011) argues that the collapse of the Communist order prompted nostalgia for an older order that could offer an alternative sense of national community. Often this has entailed invoking a distinctive national history which differentiates the former Communist states from their neighbours and which defines the Communist era as aberrant (Holy 1996). These “re-discovered” national identities routinely invoke reference to an ethnic conception of the national community in which emphasis is placed on notions of common descent and “a shared bloodline” (Mole 2011, p. 548). In turn, such conceptions of national identity have had implications for the construction of gender and sexuality. For example, if national belonging is defined in terms of “blood,” then the reproduction of national identity becomes bound up with sexual reproduction, and patriarchal gender roles are thus reproduced (Johnson and Robinson 2007). Indeed, to the degree that ethnic conceptions of the nation valorise heterosexuality, homosexuality can be conceptualised as constituting a threat to the traditional notion of the family, and thus to the nation itself.

It is important to note that not all nations routinely invoke notions of blood and lineage in their representation of the national community (Brubaker 1992; Poole 1999). Although most nations make reference to descent as a criterion of belonging, some place higher importance on alternative criteria, and it is common to differentiate between “ethnic” and “civic” forms of national belonging in which the latter is “handed out as a reward for loyalty and not on the basis of unchosen criteria such as race” (Manzo 1996, p. 19). Which criteria are adopted can impact an individual’s treatment (Hopkins et al. 2015; Wakefield et al. 2011). Moreover, these different traditions of identity construction may help explain some of the international differences in homophobia: Where the representation of the national community is not so closely bound up with biological reproduction and the ideology of the traditional family (but rather with participation in civic society), homosexuality is likely to be judged as less threatening to national identity. Indeed, in some countries a celebration of pluralism (including gay rights and culture) now forms an element of national self-definition (Mepschen et al. 2010).

## National Identification and Prejudice

Self-categorisation theory (SCT) helps to explain how our social group memberships constitute our identities (Turner et al. 1987). It purports that behaviour and attitudes are shaped by individuals’ understandings of the beliefs and values associated with their group memberships. It also suggests that individuals vary in the degree to which they invest in various groups. This means that any exploration of how national identities are implicated in homophobic prejudice must address both the individual’s understanding of the meaning of their national identity and their level of identification as a national subject.

This logic is well illustrated in research exploring people’s attitudes towards ethnic minority groups. In experimental studies which manipulate the beliefs and values associated with the nation, the treatment extended to ethnic minorities is better when the nation is defined in terms that emphasise ethnic over civic criteria (Wakefield et al. 2011). In a similar vein, survey research shows that the relationship between individuals’ level of identification with their nation and their prejudice towards migrants depends on the criteria employed in national self-definition: Stronger national identification was only associated with prejudice when the nation was defined in essentialist terms (Pehrson et al. 2009a, b).

Drawing on such findings, our research sought to explore the relationship between national identity and homophobic prejudice in two different national contexts that differ in the ways in which the nation is defined. Specifically, we gathered data in two countries that we believed would differ in the extent to which participants defined their respective nations in terms that valorised the family: Lithuania and Scotland. Following SCT’s logic that individuals’ attitudes and behaviour reflect the values and beliefs individuals associate with the social groups with which they identify, we predicted that greater identification with the nation would be associated with greater homophobia when the nation was conceptualised in terms that valorise the family.

## Lithuania and Scotland

In a synthesis of various European attitude surveys produced by Smith et al. (2014), the United Kingdom is ranked 9^th^ of 32 in terms of acceptance of non-heterosexual individuals and lifestyles, whereas Lithuania is ranked 28^th^. (For information, The Netherlands is ranked best and Latvia worst.) The United Kingdom and Lithuania can also be compared in terms of their legal policies and institutional practices and a comparative index calculated (with 100 representing complete equality for all, regardless of sexuality). Analysis by the International Lesbian, Gay, Bisexual, Trans and Intersex Association (2015) shows the United Kingdom to be at the top of this league (with a score of 86), whereas Scotland (one of the constituent countries of the UK) is at the very top (scoring 92). In contrast, Lithuania ranks 35^th^ (with a score of 19). Additional research has also confirmed that Lithuania has one of the worst records for homophobic behaviour in Europe (European Union Agency for Fundamental Rights 2012). Moreover, although Scotland is already far ahead of Lithuania in terms of sexuality-related attitudes and policies, Scotland continues to improve. Indeed, a report by the Pew Research Center (2013) illustrated the contrast between Eastern and Western Europe by foregrounding recent developments in Scotland which make it even more accepting of non-heterosexual individuals. For instance, support for same-sex marriage in Scotland has risen from 41 % in 2002 to 68 % in 2014 (ScotCen 2014), with the proportion of those who disagreed or strongly disagreed with same-sex marriage decreasing from 29 % in 2002 to 17 % in 2014. In contrast, attitudes towards the LGBT community in Lithuania appear to have worsened (Pilinkaite-Sotirovič and Žibas 2011). Based on these findings, we felt that Scotland and Lithuania were particularly suitable countries to choose for our study.

Our selection of Lithuania and Scotland was also guided by the contrasting ways in which these nations conceptualise themselves. As noted earlier, many ex-Communist countries have negotiated the need to reconceptualise their national identities through invoking conceptions of belonging which valorise the family. Lithuania illustrates this well. Indeed, discussing the Lithuanian Constitution (which as we note at the outset of our paper declares that “a family is the foundation for a society and country,” article 38.1), Kanišauskas (2012) argues that in Lithuania, the concepts of “family” and “nation” both encapsulate the idea of protecting and retaining identity and that the two terms can be used as synonyms to represent the challenge of identity-protection facing Lithuania in this post-Communist period. In turn, homosexuality is frequently defined as undermining and destroying Lithuanian family values, and, as a corollary, threatening the nation itself (Tereškinas 2007). Indeed, attempts to introduce Lithuanian civil partnerships have been defeated on the grounds that they would undermine family values (Aalia and Duvold 2012).

Such attitudes are clear in the rhetoric of Lithuania’s politicians and laws. For example, Irena Degutienė (who was the acting Prime Minister of Lithuania and also Chair of the Lithuanian Parliament) declared “we [will] never acknowledge gay marriage because it is not a real family” (Tereškinas 2007, p. 16). Moreover, legislation adopted in 2009 banned public information “that encourages [any type of] sexual relations among minors that denigrates family values or that promotes any concept of marriage and the family other than that defined in the Lithuanian Constitution and Code of Civil Law” (legislation cited in Bradley 2009: see Lietuvos Respublikos Seimas 2009).

Tereškinas (2007, p. 16) explains that such language is potent because “a fight for the family is often presented as a fight for the Lithuanian nation.” Certainly, pro-LGBT events are routinely characterised as things that “humiliate the Lithuanian nation” and Lithuanians are called upon to “defend the nation and the family” from people who are gay (Tereškinas 2007, p. 17). Many such events have attracted counter-demonstrations, and the Mayor of Vilnius (Lithuania’s capital city) refused to provide a permit to allow Lithuania’s LGBT community to celebrate “Rainbow Days 2007”—a series of events organised around the International Day Against Homophobia and Transphobia (a decision subsequently condemned by the European Commission).

Public discourse about the nation is very different in Scotland. Scottish national identity is strong, and this is reflected in campaigns for constitutional change. Yet, instead of being primarily defined in terms of lineage, Scottish national identity is frequently defined through reference to the historical distinctiveness of its civic institutions—especially its legal and educational systems that differentiate it from neighbouring England (McCrone 2002; Reicher and Hopkins 2000). In turn, Scottishness tends to be conceptualised in more civic than ethnic terms, and the family rarely features as a prominent motif in the construction of Scotland’s identity. (Indeed, where non-civic imagery is invoked, it is often in the form of reference to the land; Leith and Soule 2011.) In turn, there is little evidence for the idea that homosexuality is seen as posing a threat to the Scottish national community. For example, the Scottish Government has commended same-sex parenting for being more egalitarian than other-sex parenting, as well as for providing children with a variety of benefits (Scottish Government 2009). More recently, Scotland’s First Minister, Nicola Sturgeon, has championed moves towards increasing LGBT equality and rights (Pink Pink News 2016).

## The Present Study

For our research, we examined the degree to which strong identification with the national in-group is linked to homophobia in Lithuania and in Scotland. Given the differing conceptualisations of national identity in the two countries outlined previously, we predicted a stronger relationship between national identification and homophobia in Lithuania than in Scotland. Further, we predicted that this difference would be mediated by the extent to which participants endorsed the idea that the family lies at the heart of these different national identities.

More formally, we proposed three hypotheses. Lithuanian participants would perceive the family as being more important for their national identity than Scottish participants (Hypothesis 1a) and would hold more homophobic attitudes than Scottish participants (Hypothesis 1b). There would be a stronger relationship between participants’ level of national identification and homophobia among the Lithuanian participants than among the Scottish participants (Hypothesis 2). Perceptions of the importance of family values for the national community would mediate the relationship between national identification and homophobia, with this being moderated by whether the national identity in question is Lithuanian or Scottish. Specifically, we predicted that in Lithuania in particular, higher levels of national identification would be associated with higher levels of perceived importance of the family for the nation’s identity, which in turn would predict higher levels of homophobia (Hypothesis 3).

## Method

### Participants and Procedure

University students from Lithuania and Scotland responded to a request to participate in an online study. The link was distributed via Facebook pages associated with student organisations in Lithuania and Scotland and via personal Facebook contacts in both countries. Fully 335 individuals provided usable data (in the sense of the participants defining themselves as Lithuanian/Scottish and completing more than a minimal number of questions): 170 women, 163 men, 2 unclassified; *M*_*age*_ = 22.17, *SD* = 5.82, range = 16–60. The sample consisted of 159 Lithuanians (70 women, 87 men, 2 unclassified; *M*_*age*_ = 21.62, *SD* = 3.12, range = 16–36) and 176 Scots (100 women, 76 men *M*_*age*_ = 22.66, *SD* = 7.42, range = 17–60). Analyses revealed no significant age difference between the two national samples, *t*(239) = 1.70, *p* = .09, however, the comparisons of the gender distributions showed that the Scottish sample contained proportionately more women, *χ* ^*2*^(1) = 4.97, *p* = .026.

### Measures

All participants were presented with the same online questionnaire, but the questions were presented in Lithuanian for the Lithuanian participants and in English for the Scottish participants. The Lithuanian version was back-translated into English to ensure grammar and phrasing were correct. All items were presented on 1 (*disagree*) to 7 (*agree*) scales (and in the following order).

#### National Identification

Participants completed a four-item measure of national identification: “This national identity is very important to me”; “This national identity means little to me”[reverse scored]; “I feel proud to have this national identity”; and “This national identity has no emotional significance to me”[reverse scored]. The items were taken from previous research (Hopkins et al. 2007). The items were averaged to form a scale where higher values indicate stronger national identification (*M* = 5.34, *SD* = 1.42, α = .91).

#### Importance of Family Values

Participants were then presented with five items which measured the extent to which they perceive traditional family values as being important for the maintenance and development of their national identity: “Lithuania’s/Scotland’s future depends on having strong nuclear families (families that have mother, father and children)”; “Without strong families Lithuania/Scotland has no future”; “Family traditions are important to Lithuanians/Scots”; “Lithuanian/Scottish values are bound up with valuing the family”; and “Anything that challenges the integrity of the family will undermine Lithuania’s/Scotland’s national identity”. The items were created for our study and were averaged to form a scale where higher values indicate that traditional family values are perceived as more important for national identity (*M* = 4.06, *SD* = 1.29, α = .82).

#### Homophobia

Finally, participants’ homophobia was measured with an adapted nine-item version of the Attitudes Toward Lesbians and Gay Men Scale (Herek 1984). The original scale had 20 items designed to differentiate between several components in beliefs concerning people who are gay. Our selection was designed to tap a general sense of the acceptability of homosexuality in a short scale. The items were: “Homosexual couples should be allowed to adopt children the same as heterosexual couples” [reversed scored]; “Homosexuals should not be allowed to teach school”; “Homosexuality is a perversion”; “Homosexuality is a natural expression of sexuality” [reversed scored]; “If a person has homosexual feelings, he/she should do everything to overcome them”; “I would not be too upset if I learned that my son/daughter was a homosexual” [reversed scored]; “Sex between two same-gender people is just plain wrong”; “It sounds ridiculous that homosexuals are allowed to get married”; and “Homosexuality is another form of sexuality and should not be condemned” [reversed scored]. The items were averaged to form a scale where higher values indicate higher levels of homophobia (*M* = 2.32, *SD* = 1.47, α = .93). The study then ended and participants were debriefed.

## Results

### Between-Nation Differences

Table 1 reports between-nation comparisons on the key outcome measures. In accordance with Hypothesis 1a, a multivariate analysis of variance revealed that Lithuanian participants perceived family values to be more important for their national identity than did Scottish participants, *F*(1, 315) = 71.34, *p* < .001, ηp^2^ = .19. In accordance with Hypothesis 1b, they also expressed higher levels of homophobia than did Scottish participants, *F*(1, 315) = 128.26, *p* < .001, ηp^2^ = .29. Meanwhile, Scottish participants identified more with their nationality than did Lithuanian participants, *F*(1, 315) = 12.14, *p* = .001, ηp^2^ = .04.

**Table 1:** Descriptive statistics and correlations for study variables

Variables	Location		Correlations			
	Lithuania *M* (*SD*)	Scotland *M* (*SD*)	1	2	3	4
1. Gender (female = 1, male = 2)			–	−.25**	−.08	.18*
2. National Identification (1–7)	5.06 (1.61)	5.60 (1.19)	−.11	–	.24**	−.01
3. Importance of Family Values (1–7)	4.65 (1.29)	3.54 (1.05)	.03	.47***	–	.32**
4. Homophobia (1–7)	3.13 (1.59)	1.56 (.82)	.20*	.22**	.49***	–

Correlations for Scotland are above the diagonal; Lithuania, below

* *p* < .05. ** *p* < .01. *** *p* < .001

With regard to the above analyses, it should be noted that the measure of national identification was negatively skewed, whereas the measure of homophobia was positively skewed. Moreover, values for Levene’s Test for Equality of Error Variances were significant (*p* < .05) for each of the three outcome variables. Accordingly, we repeated the analyses, using first, Welch’s *t*-test for unequal variances, and then the non-parametric Mann-Whitney U test. The results were unchanged: on all three outcome variables there were significant (*p* < .05) differences between the Scottish and Lithuanian participants.

For completeness, we also examined the effect of gender (and nationality) on each outcome in another multivariate analysis of variance. Although women identified more with their nationality than men, *F*(1, 312) = 7.65, *p* = .006, ηp^2^ = .02, and men expressed more homophobia than did women, *F*(1, 312) = 10.71, *p* = .001, ηp^2^ = .03, there were no significant interactions between nationality and gender on any of the outcome variables (*p*s > .19), and the main effects of nationality were unaltered.

### Correlational Analyses

Pearson product-moment correlation coefficients between each of the variables described previously are also presented in Table 1. Both national samples exhibited significant positive correlations between national identification and perceived importance of family values. We also found that in both locations there was a positive correlation between the importance attributed to family values and homophobia. Most importantly (and in accordance with Hypothesis 2), we found the correlation between participants’ level of national identification and their level of homophobia was positive for the Lithuanian participants, but was non-existent for the Scottish participants. These latter results were replicated when gender was added as a covariate (Lithuanian: *r* = .25, *p* = .002; Scottish: *r* = .026, *p* = .74). We also found that Scottish participants exhibited a significant negative correlation between gender and identification (with women being more identified with the Scottish nation than were men), whereas this correlation was not significant for the Lithuanian participants. Moreover, men were more homophobic than were women in both nations.

### National Identification, Homophobia, and Family Values

Our next analysis investigated if and how beliefs concerning the importance of family values for the national community mediated the relationship between level of national identification and homophobia in the two national locations. This mediation was explored through an analysis of conditional indirect effects. This analysis allows exploration of the effect of an independent variable on a dependent variable via another variable whether or not the independent variable has a significant total effect on the dependent variable (Hayes 2009). Accordingly, we used Hayes’ PROCESS macro (Hayes 2012) which allowed us to investigate the indirect effect of national identification on homophobia through perceived importance of family values. Moreover, because each relationship could be moderated by the national location (Lithuania vs. Scotland), our analyses included location as a moderator of all relationships. The analysis involved 5,000 bootstrapping samples and 95 % confidence intervals. Because our Scottish sample included proportionately more women than the Lithuanian sample, we repeated the analysis with gender and age entered as control variables.

The output of this analysis is best described in three sections. The first concerns the relationship between participants’ level of national identification and the significance of the family for the nation. The second concerns the relationship between both level of national identification and the importance ascribed to the family with individuals’ levels of homophobia. The third (and most important in terms of our predictions) concerns the way in which valorisation of the family mediated the relationship between participants’ level of national identification and their homophobia, as well as how this mediating role depends on the nation in question (predicted to be greater in Lithuania than in Scotland).

#### Predicting Perceived Importance of Family Values

The analysis revealed that level of national identification positively predicted the perceived importance of family values (*b* = .30, *SE* = .05, 95 % CI [.21, .39]). Furthermore, location positively predicted the perceived importance of family values (with Lithuanian participants perceiving family values as more important than Scottish participants) (*b* = -1.28, *SE* = .12, 95 % CI [−1.52, −1.03]). The interaction between level of national identification and location did not predict perceived importance of family value (*b* = −.14, *SE* = .09, 95 % CI [−.32, .03]). This latter finding means that in both locations, higher levels of national identification were associated with stronger beliefs about the importance of family values for the national community (also see Table 1).

#### Predicting Homophobia

With respect to predicting homophobia, the analysis indicated that participants’ level of national identification did not predict homophobia (*b* = −.04, *SE* = .05, 95 % CI [−.14, .06]). However, the perceived importance of family values did (*b* = .43, *SE* = .06, 95 % CI [.31, .54]), as did location (with Lithuanian participants reporting higher levels of homophobia than Scottish participants) (*b* = −1.06, *SE* = .15, 95 % CI [−1.35, −.77]). Interestingly, whereas the interaction between level of national identification and location did not predict homophobia (*b* = −.07, *SE* = .10, 95 % CI [−.26, .12]), the interaction between perceived importance of family values and location did (*b* = −.31, *SE* = .12, 95 % CI [−.54, −.08]).

**Fig. 1:**
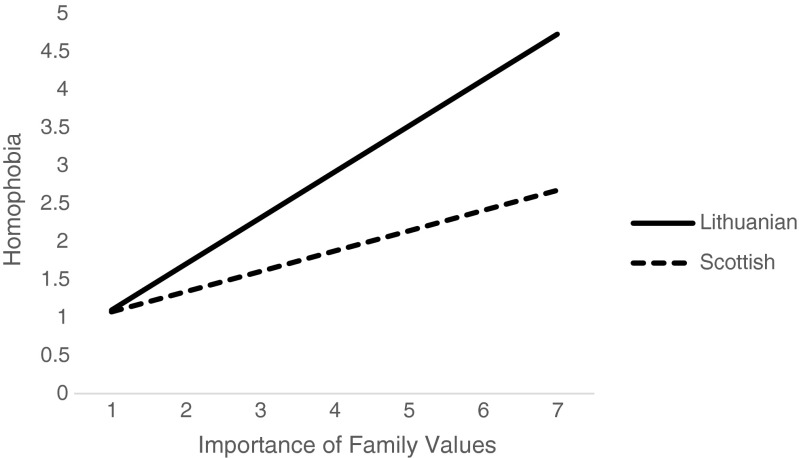
The moderating effect of location (Lithuania vs. Scotland) on the relationship between perceived importance of family values and homophobia, after controlling for national identification. All predictor variables were *Z*-scored prior to analysis

In order to examine this interaction in more detail (and thus understand how location impacted the relationship between the judged importance of family values and individuals’ levels of homophobia), we conducted a regression analysis which controlled for the effect of individuals’ level of national identification (all predictor variables were *z*-scored prior to analysis). The results were then plotted using simple slopes analysis (Preacher et al. 2003): see Fig. 1. Both the Lithuanian slope (simple slope = .78, *SE* = .10, *t* = 8.06, *p* < .001) and the Scottish slope (simple slope = .05, *SE* = .11, *t* = 3.11, *p* = .002) were significant—revealing that as the importance ascribed to family values for the nation increased, so too did homophobic attitudes. Nonetheless, the plot revealed that whereas Lithuanian and Scottish homophobia levels were similar (lower) when family values were judged as relatively unimportant for the nation, the effect of location was strong when family values were judged as relatively important for the nation. More specifically, when family values were judged as important for the nation, homophobic attitudes were stronger in Lithuania than in Scotland. Indeed, additional analyses revealed that although there was no difference between Lithuanian and Scottish participants’ homophobia levels when perceived importance of family values was low (simple slope = −.01, *SE* = .18, *t* = −.07, *p* = .95), this difference was highly significant when perceived importance of family values was high (simple slope = −1.03, *SE* = .17, *t* = −5.87, *p* < .001). It therefore seems that when family values are judged as relatively important in Lithuania, then levels of homophobia are particularly high. This implies that in Lithuania, valorisation of the family is particularly consequential for attitudes about homosexuality.

#### The Conditional Indirect Effect of National Identification on Homophobia

Bootstrapping analysis revealed that the indirect effect of level of national identification on homophobia via the perceived importance of family values was significant (*p* < .05) at both levels of the moderator (Lithuania: *b* = .22, *SE* = .05, 95 % CI [.13, .34]; Scotland: *b* = .06, *SE* = .03, 95 % CI [.02, .14]). This shows that in both locations, participants’ level of national identification had an indirect effect on their level of homophobia via the importance attached to family values. However, and as predicted (Hypothesis 3), the index of moderated mediation analysis revealed that the Lithuanian model was significantly stronger than the Scottish model (Index = −.15, *SE* = .06, 95 % CI [−.28, −.05]). Again it seems that in Lithuania, valorisation of the family is particularly consequential for attitudes towards homosexuality. These analyses in PROCESS were repeated with gender and age entered as control variables. The patterning of the results was unchanged.

#### Homophobia in the Lithuanian Sample

Because we found a direct association between level of national identification and homophobia in the Lithuanian sample (but not in the Scottish sample), we analysed the Lithuanian data separately to see if the significance attached to family values helped explain the relationship between individuals’ level of national identification and their homophobia. Specifically, we investigated whether the importance of the family for the nation fully mediated the relationship between individual’s level of national identification and homophobia. We found that it did: Whereas the total effect of level of national identification on homophobia was significant (*b* = .21, *SE* = .08, *95 % CI* [.06, .37]), the direct effect of level of national identification on homophobia (which takes into account the effects associated with the importance ascribed to family values) was not significant (*b* = −.01, *SE* = .08, *95 % CI* [−.16, .15]). Again, this pattern highlights the way in which the valorisation of the family in Lithuania explains the relationship between individuals’ level of national identification and the extent of their homophobia.

## Discussion

The findings obtained in the present study support our predictions. First, we found that Lithuanian participants perceived family values to be more important for their national identity than did Scottish participants (Hypothesis 1a), and that Lithuanian participants expressed higher levels of homophobia than did Scottish participants (Hypothesis 1b). Second, we found that the relationship between participants’ level of national identification and homophobia was stronger in Lithuania than in Scotland (Hypothesis 2). Finally, participants’ level of national identification had a stronger indirect effect on levels of homophobia mediated through the weight given to the importance of the family for national identity in Lithuania than in Scotland (Hypothesis 3). These data therefore confirm the relevance of different visions of the national community in predicting homophobia. That is, it seems the national context shapes the social significance of the family for social attitudes, and this helps explain the differing relationship between levels of national identification and homophobia in the two countries. Indeed, we found that for the Lithuanian sample the importance of the family for the nation fully mediated the relationship between individuals’ level of national identification and their homophobia.

However, it is also important to note an unexpected finding. In Scotland (where there was no *direct* association between level of national identification and homophobia), we found there was an *indirect* effect via the weight given to the importance of the family for national identity. This shows that even where at first sight the relevance of people’s level of national identification for their homophobia seems weak, on closer inspection there may be indirect relationships. Once again, this finding confirms the social significance of the nation for all manner of social attitudes (Reicher and Hopkins 2000).

Our results demonstrate the utility of SCT’s approach to group behaviour (Turner et al. 1987). Specifically, we show the utility of considering; (a) group members’ understandings of their national group membership and (b) the extent to which group members are invested in their national identity. Moreover, the current work complements existing analyses of prejudice conducted within the self-categorisation tradition. Whereas previous work has addressed the consequentiality of national definitions for attitudes towards ethnic minorities (Pehrson et al. 2009a, b; Wakefield et al. 2011), we show that such definitions also have a role to play in attitudes towards sexual minorities. However, none of the previous implies that individual difference variables are irrelevant in predicting homophobia. Indeed, there is reason to believe that contextual factors may make such variables relevant. For instance, in periods characterised by rapid social change and the public manifestation of diversity, authoritarian individuals may be motivated to express homophobic attitudes (Stenner 2005).

With regards to the situation in Scotland, it is important to emphasise that the battle for gay equality has not yet been won: Inequalities and prejudice remain. Moreover, homophobic prejudice may be associated with particular visions of the Scottish national community. Conceptions of national identity are socially constructed (rather than fixed givens), and at any one time a variety of identity formulations may be found in circulation (Reicher and Hopkins 2000). In particular circumstances, certain versions of national identity may come to the fore. However, this hegemony is far from complete, and there are always alternatives. Indeed, these alternatives may be relevant to explaining why, among our Scottish participants, we found an indirect effect of the level of national identification on homophobia via the perceived importance of family values.

### Limitations and Future Research Directions

Of course, our study is not without its limitations. For instance, some participants may have felt that it was socially unacceptable to endorse homophobic statements in the questionnaire due to social desirability concerns. However, the anonymity of the questionnaire should have helped to reduce this problem. Nonetheless, future research could perhaps measure homophobia in more subtle ways (e.g., Implicit Association Test; Greenwald et al. 1998). Additionally, it should be noted that our study involved recruiting a student sample via social media. The limitations of student samples are well-established (Henrich et al. 2010), and using social media inevitably restricts the sample to computer users with access to (and accounts on) social media websites. Future research could sample a wider demographic using alternative recruitment strategies.

Furthermore, it is important to appreciate that the analyses conducted in the present study do not allow for causal inferences to be made. Indeed, it is entirely possible that any causal relationships (if they exist) could be different from those we suggested. For example, it could be the case that the perceived importance of family values for the nation causes both high levels of national identification and high levels of homophobia. Future research could usefully explore such possibilities, ideally in longitudinal designs. Finally, it should be remembered that people who are gay are only one minority group that may experience prejudice and discrimination because of the valorisation of the family, and future research could examine the prejudice directed towards other groups that could be seen to be challenging “traditional” family structures (e.g., heterosexuals who choose not to have children). Indeed, it is likely that analyses such as ours could be applied to attitudes towards abortion (see Albanese 2004).

### Practice Implications

Perhaps most importantly, the results from the present study have implications for individuals and collectives who wish to cultivate a more harmonious and inclusive conceptualisation of the nation. Our results suggest that such people would do well to appreciate the significance of gender roles in the national imagination (Nagel 1998; Yuval-Davis 1997). Moreover, given the political relevance of the nation as a category, it is important to consider the social processes that support and sustain constructions of national identity that valorise traditional conceptualisations of the family and heterosexual normativity. As we noted, national identities and other socially significant identities (e.g., religious identities) are contested (Hopkins and Kahani-Hopkins 2004; Reicher and Hopkins 2000). This means that in any one country, at any one time, there will be a range of alternative constructions of national identity, and although one may be more hegemonic than the others, these others will be relevant for some individuals.

Campaigners need to be aware of this range of national identities, as well as the ways in which they are constructed. On the one hand, these individuals need to be aware of how others (e.g., elites concerned with managing—symbolically—various threats to their own position) produce and disseminate constructions of homosexuality as a threat to national identity (Graf 2010; Stella and Naratova 2015; van Klinken 2014). On the other hand, they need to consider the ways and means through which alternative visions of the nation can be advanced in order to challenge such heteronationalist constructions. With regard to the latter, one obvious strategy is to celebrate the lives and achievements of people who are gay in the “national story.”

Consider the case of Alan Turing. Turing revolutionised computer science and played a prominent role in the British effort to decode enemy military codes in World War II. Yet, in an era when homosexual acts were criminalised, Turing was prosecuted and underwent chemical castration as an alternative to imprisonment. He subsequently committed suicide. Recently, Turing’s achievements have received belated national recognition. Indeed, he has been re-cast as something of national hero (receiving a Prime Ministerial apology, a pardon from the Queen, and a statue commemorating his contribution to the national war effort). Celebrating the lives of “national heroes” such as Turing is one way in which activists and campaigners may promote more inclusive conceptualisations of the nation. Nonetheless, it needs to be recognised that highlighting individuals’ gender and sexuality can be problematic: Where gender and sexuality are irrelevant to an individual’s work and national contribution, references to their gender and sexuality can limit their capacity to participate on their own terms (see Sorrentino and Augoustinos 2016).

Another potential strategy is to re-present homophobia (rather than homosexuality) as incompatible with the nation’s norms and values. A particularly interesting example of this may be found in Ireland, where LGBT activists seeking to build popular support for legal reform chose to define the Irish as a “naturally” open-minded and fair people (Dunphy 1997). Campaigning under the slogan “Proud to be Irish, Proud to be Gay,” they construed Irishness as synonymous with tolerance and depicted homophobia as something that is not really “Irish,” but rather is the legacy of an alien national culture—specifically, British colonialism. This strategy thus promotes the clear message that if one wishes to be seen as Irish, one cannot be homophobic.

The success of such strategies cannot be assumed. Much will depend on the argumentative resources available in particular national communities, as well as activists’ skills in drawing upon these to bring into being new visions of the community (Hopkins and Kahani-Hopkins 2004; Reicher and Hopkins 2000). Moreover, the reception of such constructions will depend on national citizens’ relationships with the nation. Analysts differentiate between *conventional* and *constructive* patriotism, with the latter encouraging the critical reappraisal of the contemporary state of the nation (Sekerdej and Roccas 2016). This means that critical evaluations of the nation are likely to be received differently according to the type of patriotism individuals endorse.

### Conclusion

Previous psychological work has explored a range of predictors of homophobia. The present study expands upon such research by investigating the role of national identity-related beliefs. More specifically, our work provides cross-national data which shows that differing conceptions of the extent to which the nation’s identity is bound up with traditional notions of the family can be consequential for the nature of the relationship between national identification and homophobic prejudice. Our work therefore underlines the significance of these “imagined communities” (Anderson 1991) for gender-role research, for homophobia, and for political and social intervention.
